# Psychosocial and Motor Characteristics of Patients With Hypermobility

**DOI:** 10.3389/fpsyt.2021.787822

**Published:** 2022-03-28

**Authors:** Mateus M. Lamari, Neuseli M. Lamari, Gerardo M. Araujo-Filho, Michael P. Medeiros, Vitor R. Pugliesi Marques, Érika C. Pavarino

**Affiliations:** ^1^SService of Physiotherapy, Fundação Faculdade Regional de Medicina de São José do Rio Preto, FUNFARME, São José do Rio Preto, São Paulo, Brazil; ^2^Department of Neurological Sciences, Psychiatry and Medical Psychology, Faculdade de Medicina de São José do Rio Preto - FAMERP, São José do Rio Preto, São Paulo, Brazil; ^3^Graduated Student, Department of Neurological Sciences, Psychiatry and Medical Psychology, Faculdade de Medicina de São José do Rio Preto - FAMERP, São José do Rio Preto, São Paulo, Brazil; ^4^Service of Neurology, Santa Casa de Misericórdia de São Carlos, São Carlos, São Paulo, Brazil; ^5^Department of Molecular Biology, Faculdade de Medicina de São José do Rio Preto - FAMERP, São José do Rio Preto, São Paulo, Brazil

**Keywords:** Ehlers-Danlos syndrome, pain, joint hypermobility, articular instability, fatigue, anxiety, psychosocial

## Abstract

**Objectives:**

To identify psychosocial and motor aspects related to joint hypermobility (JH) in a sample from almost all Brazilian states by age range and sex; to characterize JH by the Beighton total score ≥4, ≥5, and ≥6 according to sex and age and atypicality in the sitting position and in the hands; identify, in the total sample, manifestations of “growing pain” and its location, fatigue, attention deficit, anxiety, insomnia, drowsiness, apathy, depression, delay in walking, not crawling or crawling differently, school performance, spatial orientation and/or temporally impaired, social isolation, and being stigmatized as “lazy/clumsy/apathetic”.

**Methods:**

This retrospective, observational, quantitative, and cross-sectional study used data obtained through analyses of descriptive and inferential crossings between 2012 and 2020 of 482 medical records of individuals between 1 and 76 years of age, from most Brazilian states. All patients previously diagnosed with “joint hypermobility syndrome” (JHS) and “Ehlers-Danlos syndrome hypermobility type” (EDS-HT) had their medical records reassessed, following the guidelines established in 2017. The analysis of GJH was performed using the updated method by Beighton method; atypical characteristics were investigated in the hands and the ability to sit in the “W” and the “concave” positions. The characteristics and manifestations of “growing pain” and its location were analyzed in the total sample, fatigue, insomnia, drowsiness, apathy, depression, social isolation, attention deficit, anxiety, stigmatization as “lazy,” clumsy/restless, impaired school performance, and spatial and/or temporal orientation. Descriptive and inferential statistical methods were used, such as Mean, Median, Mode, Standard Deviation, Standard Error, Maximum Value, Minimum Value, *Komolgorov-Smirnov*, Significance, Relative Value, Absolute Value, *Mann-Whitney U*, and Correlation of *Spearman*.

**Results:**

JH in the total sample predominated in the upper limbs, the majority were women, represented by 352 (73.02%), 15 years old or older with 322 (66.80%), 312 (64.73%) had a Beighton total score ≥6, which decreased as the age increased. Always sitting in the “concave” position was represented by 54.15% and the ability to sit in the “W” position by 39.21%; signs on the hands totaled between 27.59 and 44.19% with a significant correlation between the variables. Among the characteristics, fatigue predominated, followed by an awkward/clumsy/restless individual, attention deficit, anxiety and stigmatized as “lazy,” insomnia, drowsiness, apathy, depression, impaired spatial and/or temporal orientation, and social isolation. From the total sample, pain in the lower limbs was reported by 55.81% and having or having had “growing pain” was reported by 36.93%, delay in walking occurred in 19.92%, 15.35% did not crawl or crawled differently, and for 12.86%, school performance was impaired. Higher Beighton total scores showed a trend towards motor implications and correlation between variables. Ability to still sit in the “concave” position was possible for 54.15% and to sit in the “W” position for 39.21%.

**Conclusion:**

In the total sample, the JH characteristic prevails in the upper limbs of female children, adolescents and adults, with a total Beighton score ≥6. Most sit in the “concave” position and less than half also sit in the “W” position and with atypical hand postures. The higher Beighton scores, which include the upper limbs, show a tendency to not crawl or crawl differently, delayed ambulation, and impaired school performance. The predominance of JH in the upper limbs is suggestive of a justification for not crawling or crawling differently. Characteristics of atypical motor performance in hands and sitting posture, in addition to fatigue, pain since childhood, anxiety, apathy, depression, sleep disorders, stigmatization, attention deficit, spatial and/or temporal orientation impairment, and social isolation are characteristics. suggestive of psychosocial implications at different ages. Future studies with motor and psychosocial aspects of people with JH will help to identify the phenotype of this population and consequent guidance for clinical management based on the motor and psychosocial aspects of people with JH.

## Introduction

### Joint Hypermobility

Joint hypermobility (JH) may represent the extreme normal range of the motion spectrum or condition for a group of hereditary connective tissue disorders, which is influenced by age, sex, and ethnicity (1). It has a Gaussian distribution on the spectrum of physiological range of motion and is considered a genetically determined deviation from normality (2). It can be a descriptor and exist as an isolated diagnostic finding, but it is often a feature of a larger syndromic diagnosis, rarely diagnosed (3), and considered by most professionals as a harmless ability (4).

The method for JH assessment was proposed by Beighton et al. (5). In 2017, an International Consortium for the Ehlers-Danlos Syndromes (EDS) proposed the 2017 International Classification for EDS and recognized 13 subtypes of EDS (2). Among the 13 subtypes, the hypermobile EDS subtype is similar to the hypermobility spectrum disorders (HSD), an underrecognized group of connective tissue disorders that involves a spectrum ranging from asymptomatic hypermobility, or hypermobility affecting only one joint, to generalized joint hypermobility (GJH) (6). This new classification included the Beighton method among its diagnostic criteria, with adjustments, and considerations that included changes in the parameters of the scores. The score ≥6 is for prepubertal children and adolescents, a score ≥5 is for pubertal men and women up to 50 years of age, and a score ≥4 is for those over 50 years of age (2). In the same year, Castori et al. (6) proposed a criteria for identification of the characteristics.

The diagnosis for hypermobile Ehlers-Danlos syndrome (hEDS) is clinical and considers the presence of criteria (6, 7) including a spectrum of phenotypes, ranging from asymptomatic condition, non-syndromic JH, to hEDS, and HSD. In 2019, Copetti et al. (8) explored the clinical ramifications of hEDS and HSD and grouped these conditions into a single phenotype termed hEDS/HSD and considered severity in class distinction. HSD has become updated diagnostics for all those individuals who were diagnosed by previous criteria as hEDS-HSD or joint hypermobility syndrome (JHS). HSD is also intended to identify milder subtypes that fill the entire gap between asymptomatic JH and hEDS (6).

GJH is relatively common, with a prevalence of 2–64.6% in different populations, varying among age, sex, and ethnicity of the population (9–11). It is common in childhood, affecting ~8–39% of school-age children (12), 41% of children and adolescents (13), and 64.6% of pre-school-age children (9). It is more frequent among women and children and among Asians, followed by African-Americans and Caucasians (14–16). However, the locomotor system of the child with JH, already in the first year of life, may present characteristics that signal implications in neuropsychomotor development (17–19). Signs may include motor and cognitive deficits, benign motor delay (20), frequent falls (21–24), “flying bird” hand sign, Steinberg sign, and Walker-Murdoch sign (19, 25–27).

Individuals with JH are more vulnerable to microtraumas (28–30) and macrotraumas (31), and eventually adopt inadequate postural habits (32–34) because of their less robust tissues, which imply insufficient support of the physiological function of the locomotor apparatus. It is relevant that tissues can suffer degenerative processes (35), with insidious and silent signs and symptoms (36). This pattern of joint involvement results in several well-defined physical factors and implications in other characteristics resulting from inadequate postures (37).

In addition to motor characteristics, psychological dysfunctions and emotional problems, including depression, anxiety, affective disorder, low self-confidence, negative thoughts, hopelessness, and despair, are common among these patients (38–41).

### Psychosocial Disorders Related to Hypermobility

Psychological dysfunctions and emotional problems are common among those with EDS and may have consequences for ignoring or not confronting the presence of comorbid psychological problems, which may lead to suboptimal treatment (42). However, there is a growing amount of evidence pointing to a high prevalence of psychiatric conditions among individuals with EDS (41), which includes, among others, anxiety disorders, depression, and neurodevelopmental disorders, such as attention deficit hyperactivity disorder, and other clinical manifestations, associated with JH (43, 44).

Difficulties in the acquisition and performance of motor skills results in categorization of this population as clumsy, stubborn, and with clumsy handwriting, poor postural control, and difficulties in motor learning. Study by Vaivre-Douret (45) corroborates these results, pointing out that children with developmental coordination disorders are characterized by difficulties in the acquisition and performance of motor skills, which persist into adolescence and adulthood.

A previous study reports signs of malaise in these patients, lack of concentration and memory, low self-efficacy and self-worth, and anxiety and depression (46). Depressive feelings are common in hEDS and can be understood as secondary to the difficulties related to the disease (47). Patients with hEDS often suffer from anxiety disorders, and the link between these two variables has been repeatedly found in the literature (48, 49). In 2021, De Vries et al. (50) showed that adolescents and young adults with a combination of GJH and anxiety were significantly impaired, with decreased physical and psychosocial functioning, decreased workload, increased fatigue, and debilitating pain (50).

Fatigue is also present in most affected individuals, manifesting as persistent feelings of tiredness, lack of energy, and feelings of exhaustion with impaired concentration (51). Bravo (26) found symptoms of chronic fatigue, dizziness, and fainting in 40% of men and 64% of women <30 years of age, a condition that receives little medical attention (52).

In general, people with EDS report how they must deal with the social image that people around them have about them. They are labeled as lazy, apathetic, and tired people who are unable to accomplish a project, when in fact they are too exhausted to do it (53). In addition, because of the invisible nature of this disease, they face judgments from friends, family members, and strangers (54). Thus, some patients exhibit an excessive rest pattern. Therefore, they are labeled hyperactive or lazy, or still as unsociable, and depressed (47). Shame, guilt, and stigma may have negative psychosocial consequences, decrease self-esteem, and lead to depression (54) and feelings of social isolation (55).

Nocturnal insomnia and morning sleepiness are frequent in hEDS (56–58). Patients with hEDS report sleeping problems, including insomnia and non-restorative sleep (14, 59). Baeza-Velasco et al. (39) cite that reduced sleep is related to pain complaints, with psychosocial consequences and chronicity for the quality of life. The presence of “growing pain” in children was reported by Matsudo et al. (60), who identified that in most cases pain occurred in the lower limbs. Early recognition and adequate treatment of problems such as sleep function, mobility, chronic pain, and psychological conditions are important for successful holistic treatment of patients with EDS (61).

## Objectives

To identify psychosocial and motor aspects related to JH in a sample from almost all Brazilian states by age range and sex; to characterize JH by the Beighton total score ≥4, ≥5, and ≥6 according to sex and age and atypicality in the sitting position and in the hands; identify, in the total sample, manifestations of “growing pain” and its location, fatigue, attention deficit, anxiety, insomnia, drowsiness, apathy, depression, delay in ambulation, not crawling or crawling differently, school performance, spatial orientation and/or temporally impaired, social isolation, and being stigmatized as “lazy/clumsy/apathetic.”

## Methods

### Ethical Considerations

According to the Regulatory Standards for Research in Human Beings, Resolution 466/2012 of the National Health Council, this study was approved by the Research Ethics Committee (CEP) of the São José do Rio Preto Medical School (FAMERP), Opinion CAAE No. 36145820.6.000.5415. Data collection was performed after approval of the waiver for the need of the Informed Consent Form.

This is a retrospective, observational, quantitative, and cross-sectional study. Clinical records of patients with JH, seen at the Clínica de Fisioterapia Lamari Ltda of São José do Rio Preto—SP, were analyzed. Inclusion criteria were adopted for medical records that contained the data listed in the data collection instrument.

This study was conducted after the publication of the new nosology for EDS and disorders associated with hypermobility in 2017 (2), which considers specific clinical presentations for EDS subtypes and introduces the concept of hypermobility spectrum (6). Therefore, all patients previously characterized or diagnosed with JHS/EDS-HT, according to the previous nosology and criteria, had their records reevaluated, following the guidelines established by the new nosology for EDS.

The records were made in an instrument with a specific questionnaire for the analysis of the characteristics of joint hypermobility (JH) and related clinical features. The data were tabulated in an *excel spreadsheet*, prepared as the recording tool for the study.

The results were obtained from the analyses by descriptive and inferential crossings of the data from the sample of 482 medical records of individuals seen in the period from 2012 to 2020, from 21 Brazilian states and the Federal District, totaling 124 cities. The group consisted of individuals with age group between 1 and 76 years, with a mean age of 25 years (SD = 15.94). Of these, 322 (66.80%) were aged ≥15 years, and 352 (73.02%) were women. The absolute and percentage distribution of the total sample by age-group and sex is shown in Table 1.

**Table 1:** Absolute and percentage distribution of the total sample by age group and gender.

**Age group**	**F**		**M**		**Total**	
**(years)**	**N**	**%**	**N**	**%**	**N**	**%**
1–9	54	15.34	41	31.54	**95**	**19.71**
10–14	35	9.94	30	23.08	**65**	**13.49**
15–50	230	65.34	56	43.08	**286**	**59.34**
>50	33	09.38	3	02.31	**36**	**7.47**
Total	**352**	**100.00**	**130**	**100.00**	**482**	**100.00**

The family history of JH was reported by 382 (79.25%), and others were unaware of any history. The total sample consisted of 427 (88.5%) individuals with HDS and 55 (11.4%) with hEDS.

### Data Collection Tools

Data collection was made by the researcher using a self-developed instrument from March 2020 to July 2020, to record the data obtained from the medical records of the patients seen at the Clínica de Fisioterapia Lamari Ltda of São José do Rio Preto in the period from January 2012 to March 2020. The data from the clinical history, general and specific physical examination, characteristics associated with JH, analysis by the Beighton method (5), considering the new classification criteria (2, 6), and the family history of JH were included.

### Analysis of GJH by the Beighton Method

Joint mobility was evaluated in the joints of five body regions: fifth finger, wrist, elbow, knee, and trunk, using the method proposed by Beighton et al. (5).

The Beighton total score consists of five variables, of which four are tested bilaterally and one test evaluates the lower back and lower extremities. For scoring, the following variables were considered: thumb apposition, fifth finger extension, elbow extension, knee extension, and “complete anterior flexion of the trunk with flat hands on the floor, with extended knees” (AFT). Total scores ranged from 0 to 9. Joint mobility was evaluated according to the scores proposed by Beighton et al. (5). One point was scored for each positive result (for each side), and a total score was attributed by adding up the variables. The correlation and significance between each of the Beighton scores ≥4, ≥5, and ≥6 were analyzed, according to sex and age range.

### Analysis of Hip and Trunk JH

**Figure 1:**
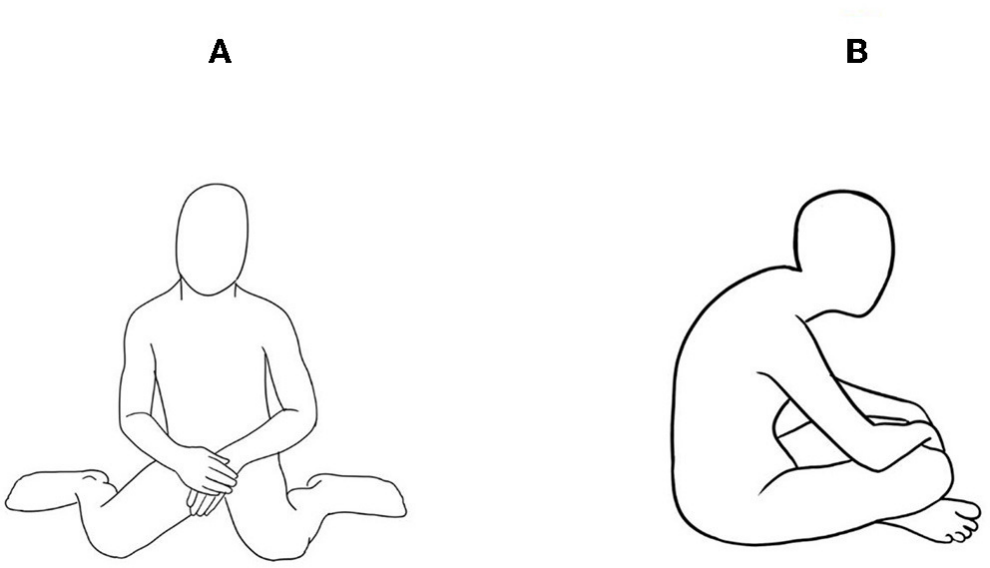
Illustration of the seated in “W” position **(A)** and concave seated position **(B)**.

The JH of the hip and trunk was evaluated using sitting positions on the ground and was defined as present when the following were observed: sitting in the “W” position with the lower limbs abducted, and with the knees in full flexion and the feet lateralized, sitting in a “concave” position, that is, trunk and head anteriorized, with protruding shoulders and abdomen. The variables sitting in “W” and “concave” positions were analyzed by “age range” and in the total sample, considering those who were never able to sit in these positions, as well as those who were always able to sit in these positions and those who had been able to sit in these positions only in the past. The illustrations are shown in Figure 1. The significance of these variables was analyzed, considering age groups, as well as in the total sample. The data are presented in **Table 5**.

### Analysis of JH Features on the Hands

**Figure 2:**
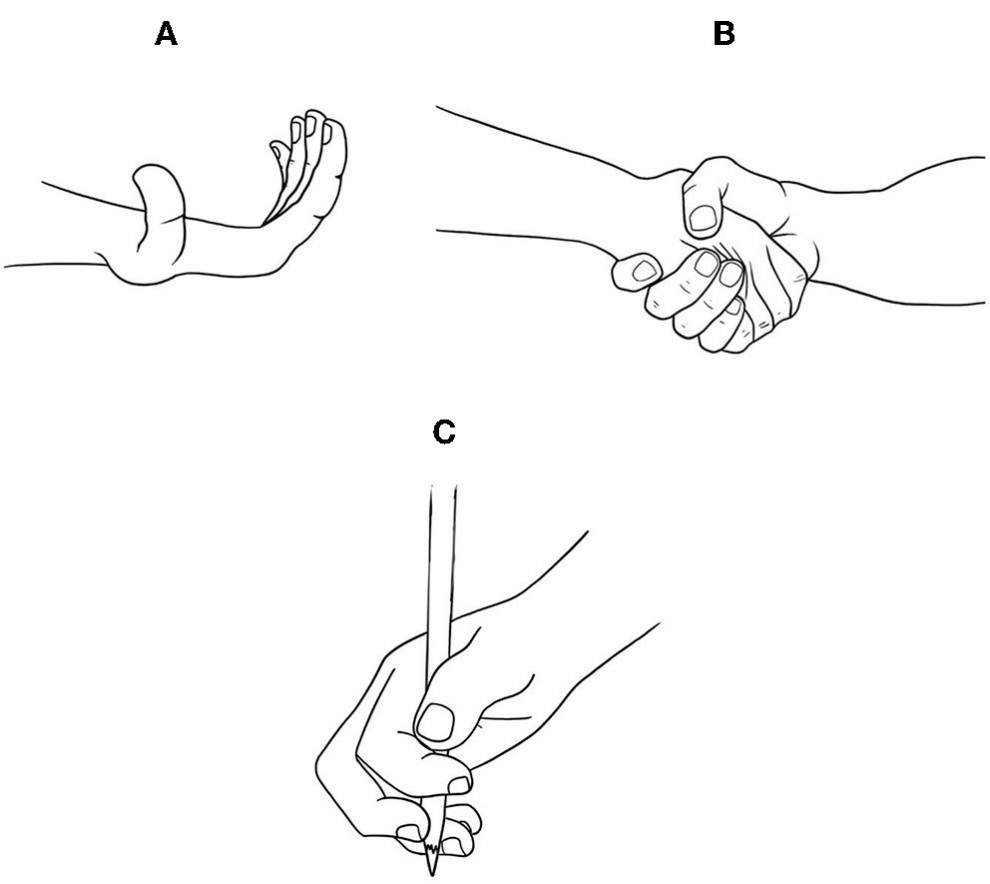
Illustration of the flying bird hand **(A)**, greeting sign **(B)**, and adaptation for writing **(C)**.

JH in the hands was defined as present when the related abilities were identified. The “birds flying” hand sign was evaluated by the active hyperextension of the fingers. The JH was defined as present when the angle of the metacarpophalangeal joints was greater than 20° in both hands. The “greeting” sign was evaluated considering the act of shaking hands when greeting. JH was defined by the perception of excessive hand mobility, as well as the sensation of softness of the skin while shaking hands during the act of greeting. Writing adaptation was evaluated during the act of writing. JH was defined as present when the patient held the pencil or pen in an atypical way, that is, not consistent with support by the pinch movement of the thumb and index fingers. The correlation and significance between these variables were analyzed. The illustrations are shown in Figure 2.

### Statistical Analysis

Descriptive and inferential statistical methods were used. Probability issues of a population were analyzed based on the sample data, for which mean, median, mode, standard deviation, standard error, maximum value, minimum value, *Komolgorov-Smirnov* test, significance, relative value, absolute value, *Mann-Whitney U* test, and *Spearman* correlation test. Hypothesis tests were performed using *Mann-Whitney's U* test and *Spearman*'s correlation test to analyze the behavior of correlations between the variables analyzed and the degree of explanation of the dependent variable in relation to the independent variables of the sample. The data were replicated in absolute and relative forms in this first part. In the inferential scope, it was outlined as a statistical objective, and the analysis of independence and prediction between the variables was proposed in the scope of work. The results of independence between the proposed variables were given by analysis between the values of *p* (significance). All analyses were conducted using the *SPSS Statistics Software* (version 23) linked to the features of the Excel tool (version 2.016).

## Results

### Characteristic of JH by Beighton Total Score, Sex, and Age in the Total Sample

Analyses of the total sample by Beighton scores, which scores and characterizes JH currently in five regions of the body, with a score variation of 0–9, showed that the mean was represented by score 6 (SD = 2.12). Analyses of the total sample by the Beighton scores, which scores and characterizes JH currently in five body regions, with a score range from 0 to 9, showed that the mean was represented by a score of 6 (SD = 2.12). Analyses of the distribution of the total Beighton scores in the total sample showed that the total Beighton score ≥4 was completed by 426 (88.38%), the score ≥5 by 344 (71.37%), and the score ≥6 by 312 (64.73%). The score ≤3 was filled by 56 (11.64%).

The *Komolgorov-Smirnov* Normality *and* Spearman Correlation statistical tests were applied, which pointed out that the total Beighton scores as a function of sex and age in the total sample show a non-significant tendency for women to obtain higher Beighton score scores (rs = −0.050, *p* = 0.259). Smaller stature and younger age show a tendency for higher total Beighton scores, with a statistically significant difference (rs _=_ −0.161, *p* = 0.000; rs = −0.216, *p* = 0.000).

### Beighton Scores ≥4, ≥5, and ≥6 by Sex and Age Group in the Total Sample

Analyses of the distribution of Beighton's total scores ≥4, ≥5, and ≥6 in the total sample as a function of sex are presented in Table 2, where the correlations between each of the scores ≥4, ≥5, and ≥6 with sex and their respective “*p*” values are shown. In Table 3, correlations between each of the scores ≥4, ≥5, and ≥6 with age group and their respective “*p*” values are shown.

**Table 2:** Absolute and percentage distribution of Beighton scores ≥4, ≥5, and ≥6 by gender in the total sample.

**Gender**	**F**		**M**		**Total**	
	**N**	**%**	**N**	**%**	**N**	**%**
Beighton score ≥4	312	73.24	114	26.76	**426**	**100**
Beighton score ≥5	256	74.42	88	25.58	**344**	**100**
Beighton score ≥6	227	72.76	85	27.24	**312**	**100**

*Beighton score ≥4 rs = −0.056; p = 0.248*.

*Beighton score ≥5 rs = −0.028; p = 0.601*.

*Beighton score ≥6 rs = −0.110; p = 0.053*.

**Table 3:** Absolute and percentage distribution of Beighton scores ≥4, ≥5, and ≥6 by age-group and in the total sample.

**Age**	**Upto 1–9 years**		**10–14 years old**		**15–50 years old**		**>50 years old**		**Total**	
	**N**	**%**	**N**	**%**	**N**	**%**	**N**	**%**	**N**	**%**
Beighton score ≥4	93	21.83	58	13.61	248	58.21	27	06.34	**426**	**100**
Beighton score ≥5	77	22.19	51	14.00	201	57.92	18	05.19	**347**	**100**
Beighton score ≥6	74	23.72	49	15.71	173	55.45	16	05.13	**312**	**100**

*Beighton score ≥4; rs = −0.114; p = 0.019*.

*Beighton score ≥5; rs = −0.122; p = 0.024*.

*Beighton score ≥6; rs = −0.041; p = 0.474*.

### General Physical Characteristics of JH in the Locomotor Apparatus in the Total Sample

Analyses of physical characteristics in the locomotor apparatus showed that a minimum of 133 (27.59%) and a maximum of 213 (44.19%) individuals presented different physical signs in the hands, and 261 (54.15%) participants were sitting always in the “concave” position. A majority of 337 (69.92%) participants reported that they were able to “sit in a concave position,” including those who were able to do so in the past. Sitting in the “W” position was always possible for 189 (39.21%), and when we included those who were able to do so in the past, they comprised a majority of (55.81%) of the total sample.

### Physical Signs of JH on the Hands in the Total Sample

Among the characteristics of physical signs on hands, the sign “flying bird hands” comprised 213 (44.19%) individuals, “compliance” sign, 189 (39.21%), writing adaptation, 161 (33.40%), weak hands for handling, 157 (32.57%), and tiredness due to writing, 133 (27.59%).

Analyses were performed to verify a correlation between the total Beighton score, “birds flying” sign, and the “compliance” sign in the total sample. Analyses of the “compliance” sign variable showed positive and significant correlations between the Beighton score and the “compliance” sign variable (rs = 0.167; *p* = 0.000), as well as with the “bird hands flying” variable (rs = 0.150; *p* = 0.001). Thus, the presence of the “greeting” sign and/or “flying bird hands” correlated with the Beighton scores for GJH.

Analyses to verify the correlation between the variables referring to the clinical signs of the hands are presented in Table 4.

**Table 4:** Analysis of the correlation between physical signs of JH in the hands.

**Features**	**Correlation**	***p***
Flying bird hands” sign and “greeting” sign	0.389	0.000
Hand signals “flying birds” and adaptation for writing	0.194	0.000
Signal “flying bird hands” and weak for handling	0.202	0.000
Sign “flying bird hands” and tiredness to write	0.189	0.000
Sign of “greeting” and adaptation for writing	0.206	0.000
Sign of “compliance” and weak for handling	0.249	0.000
Sign of “compliance” and tiredness to write	0.208	0.000
Adaptation for writing and weak for handling	0.681	0.000
Adaptation to writing and writing fatigue	0.685	0.000
Weakness to handle and tiredness to write	0.769	0.000

### Body Postures and Skills Never Presented, Always Presented, Presented Only in the Past, by Age-Group and in the Total Sample

The analyses show that sit “concave” was always possible for 261 (54.15%) individuals of the total sample. When also considering those who were able to do so in the past, they represented the majority with 337 (69.92%), as the difference was not significant in the total sample (*p* = 0.556) and by age-group (*p* = 0.502). The ability to sit in a “W” posture was always possible for 189 (39.21%) of the total sample. When also considering those who were able to sit in a “W” posture in the past, they represented the majority with 269 (55.81%) of the total sample, with difference not significant in the total sample (*p* = 0.429) and in the age-group (*p* = 0.284). The absolute and percentage frequencies of body postures and skills in the age-groups never presented, always presented, and presented only in the past by age-group and in the total sample and *p* values of the variables in the age-groups and in the total sample are presented in Table 5.

**Table 5:** Absolute and percentage frequencies of body postures and skills never presented, always presented, and presented only in the past by age-group and in the total sample and *p* values.

**Variable**	**Period**			**Age group**						**Total**		***p***
		**1–9 years**		**10–14 years**		**15–50 years**		**>** **50 years**				
		**N**	**%**	**N**	**%**	**N**	**%**	**N**	**%**	**N**	**%**	
Sitting concave	Never	28	29.47	22	33.85	84	29.37	11	30.56	**145**	**30.08**	0.502
	Always	55	57.89	34	52.31	152	53.15	20	55.56	**261**	**54.15**	
	In the past	12	12.63	9	13.85	50	17.48	5	13.89	**76**	**15.77**	
	**Total**	**95**	**100.00**	**65**	**100.00**	**286**	**100.00**	**36**	**100.00**	**482**	**100.0**	
	*p* = 0.556										
Sitting in “W”	Never	37	38.95	30	46.15	128	44.76	18	50.00	**213**	**44.19**	0.284
	Always	39	41.05	21	32.31	117	40.91	12	33.33	**189**	**39.21**	
	In the past	19	20.00	14	21.54	41	14.34	6	16.67	**80**	**16.60**	
	**Total**	**95**	**100.00**	**65**	**100.00**	**286**	**100.00**	**36**	**100.00**	**482**	**100.0**	
	*p* = 0.429										

### Psychosocial Characteristics in the Total Sample

Among the characteristics of psychosocial implications, fatigue accounted for more than half of the total sample. Clumsy, stubborn, or apathy individuals and individuals with attention deficit accounted for slightly less than half of the total sample. Anxiety, having been stigmatized as “lazy,” insomnia, sleepiness, social isolation, apathy, depression, and impaired spatial and/or temporal orientation comprised 15.76–28.01% of the participants. An analysis of the total sample referring to pain in the lower limbs as a symptom was reported by the majority and “growing pain” by more than one third of the total sample. The absolute values and percentages are presented in Table 6.

**Table 6:** Absolute values and percentages of psychosocial characteristics in the total sample.

**Characteristic**	***N***	**%**
Pain in the lower limbs	269	55.81
Fatigue	263	54.56
Stigmatized clumsy/stubborn/restless	194	40.25
Attention deficit	181	37.55
“Growing pains”	178	36.93
Anxiety	135	28.01
Stigmatized “Lazy”	123	25.52
Insomnia	122	25.31
Social isolation	109	22.61
Apathy	92	19.09
Sleepiness	89	18.46
Depression	80	16.60
Impaired spatial and/or temporal orientation	75	15.76

Analyses to verify in the total sample if there is a correlation between the total Beighton score and “growing pain” to show that the more the total Beighton score increases, there is a tendency not to have “growing pain,” and the difference was not significant (*p* = 0.827), according to Spearman's test (rs = −0.010).

### Aspects Related to Neuropsychomotor Development

Analysis of characteristics related to neuropsychomotor development showed that 19.92% showed delayed walking, 15.35% did not crawl or crawled differently, and school performance was impaired for 12.86%. Variables related to motor performance show that for higher total Beighton scores, there is a tendency for motor implications and correlation between the variables. Analyses to verify whether there was a correlation between total Beighton scores and having crawled differently show that as the total Beighton score increased, there was a greater tendency to crawl differently; however, the difference was non-significant (*p* = 0.548), according to the Spearman's test (rs = 0.027).

Analysis also showed that the higher the total Beighton score, the greater the tendency not to crawl, with the difference being non-significant (*p* = 0.954), according to the Spearman test (corr = −0.003). For those who crawl differently, there was a tendency to have impaired school performance, and the difference was significant (*p* = 0.036), according to the Spearman's test (Corr = 0.095); for those who crawl differently, there was a tendency to have delayed ambulation, and the difference was not significant (*p* = 0.190), according to Spearman's test (Corr = 0.060). Participants who did not crawl showed a tendency to have impaired school performance, with a non-significant difference (*p* = 0.064), according to Spearman's test (Corr = 0.084); those who did not crawl showed a tendency for delayed walking, with significant difference (*p* = 0.003), according to Spearman's test (Corr = 0.136), and for those whose school performance was impaired, there was a tendency for delayed walking, with significant difference (*p* = 0.014), according to Spearman's test (Corr = 0.112). The values are presented in Table 7.

**Table 7:** Correlation analysis between motor performance variables, Beighton score, impaired school performance, and motor performance variables.

**Features**	**Correlation**	***p***
Total Beighton score and crawl different	0.027	0.548
Total Beighton score and not to crawl	−0.003	0.954
Crawl different and impaired school performance	0.095	0.036
Crawl different and delayed ambulation	0.060	0.190
Not to crawl and impaired school performance	0.084	0.064
Not to crawl and delayed ambulation	0.136	0.003
Impaired school performance and delayed ambulation	0.112	0.014

## Discussion

Although the process of joint movement in the human species is complex, with few references to normality standards, JH seems to sensitize scholars, which can be verified by the large number of publications on the subject found in the specialized literature and with emphasis on conditions related to JH. Analyses of this study included both genders and the different age-groups including participants from almost all the Brazilian states and the Federal District and show that the characteristic of JH predominates in the upper limbs, decreases with increasing age, with most children, adolescents, and adults having a total Beighton score ≥6. However, this reference score ≥6 was proposed by the new nosology (2) only for prepubertal children and adolescents.

In this context, the prevalence of JH in the upper limbs in the total sample, predominant signs in the hands, verification of significant correlations between total Beighton score, “flying bird hands” sign, “compliance” sign, adaptation for writing, weak hands for handling, and tiredness due to writing must be considered. These characteristics in the hands, among others, such as Steinberg's sign and Walker-Murdoch's sign (25), can justify inadequate neuropsychomotor development, with motor and cognitive deficits, benign motor delay (20), and frequent falls (21–24). The “flying bird hands” sign was cited by Lamari et al. (19) and Bravo (26).

It is also emphasized by the fact that most of the individuals in the present study are capable of always sitting in the “concave” position, due to weakness, mainly, of the abdominal and paravertebral muscles. When considering this capacity only, in the past, they totaled 69.92% of the total sample. The ability to sit in the “W” position also stands out, because of hypermobility of the hips, which has always been possible for a little less than half of the total sample, and when considering also those who in the past were capable of sitting in the “W” position, they represented the majority of the total sample.

All these findings together may contribute and corroborate to explain the fact that part of this study population is not able to crawl or has crawled differently, as well as the delay in ambulation and the presence of fatigue since the beginning of neuropsychomotor development. These conditions may, in part, explain the psychosocial implications, such as anxiety, stigmatization as “lazy,” sleep disorders, apathy, impaired spatial and/or temporal orientation, depression, and finally, social isolation. The composition of the tissues of the locomotor system, along with the insufficient condition of its biomechanics, is suggestive of insufficiency for the daily demands.

It is essential to identify this population from childhood, because the locomotor system of the child with JH may present clinical signs in the first year of life (17–19). In 2005, a study with 1,120 Brazilian children aged 4–7 years of both sexes was carried out to identify hypermobile children (9). It was found that most preschool children obtained a Beighton score ≥4 (64.6%), score ≥5 by 38.2%, and ≥6 by 27% of them. The study concluded that the parameter related to score ≥4 was used for children and that other parameters should be differentiated for children. Further, inclusion of many studies could have compromised results because they included different ages in the same sample and analyzed by the same criteria and parameters. In 2017, an International Consortium for EDS proposed the 2017 International Classification for EDS and recognized 13 subtypes of EDS with secondary diagnostic criteria described for each subtype (2). Among the 13 subtypes, there is the subtype hypermobile EDS, similar to HSD, an underrecognized group of connective tissue diseases that involves a spectrum ranging from asymptomatic hypermobility or hypermobility affecting only one joint to GJH.

Another study by Yazgan et al. (62) with children investigated the prevalence and characteristics of JH in prepuberty with JH defined using the Beighton criteria. The prevalence of the characteristics with scores ≥4 was 39.3%, ≥5 with 22.7%, and ≥6 with 13.3%. The difference in the age-groups between the study populations of Lamari et al. (9) and of Yazgan et al. (62) may have accounted for the significant reduction in the presence of the JH traits that corroborate the process of tissue maturity with a decrease in joint mobility and definition of the hypermobile population even in childhood.

Morris et al. (63) analyzed JH in a cohort study of 1,584 Australian adolescents using the Beighton scoring system. The prevalence of AGH was 60.6% and 36.7% among girls and boys, respectively, considering the Beighton score ≥4; when defined as ≥6, it was 26.1 and 11.5%. The high prevalence rates of GJH as defined by the commonly used Beighton cut-off values in this cohort highlight the need to question the appropriateness of these cut-off points in future studies and be powered for sex-specific analyses due to the different prevalence rates of JH in male and female samples.

Analyses of the present study corroborate the literature on the effects of biological aging of the tissues that contribute to diminishing the JH and conceal joints affected by JH. The effect of age can negatively impact the range of motion of the joint, which has led some researchers to hypothesize the occurrence of chronic musculoskeletal symptoms in adults who progressively lose the JH. The five-point questionnaire was introduced as a rapid screening tool to investigate historical JH in adults who presumably lost GJH (64). Castori et al. (65) supported this hypothesis. This questionnaire is simple and a reproducible self-report that can be used as an adjunct in the clinical evaluation of chronic and diffuse pain syndromes where JH is often missed, as well as for individuals who have progressively lost GJH. However, it does not nominally include the characteristics observed in clinical practice, which would facilitate their historical identification, a condition that would adequate the targeted treatment. In this context, the present study investigated the physical characteristics both nominally and individually. It was observed that majority of the study sample sat concave or in the “W” position. These postures and body skills are frequent in just under half or more than half of the different age-groups. They are antifunctional and anti-aesthetic postures and can compromise the conformation of the joints and consequently the mechanics of the locomotor system and facilitate falls.

Hershenfeld et al. (41) identified psychosocial impairment in hypermobile individuals, and Tinkle et al. (42) pointed out psychological dysfunction and emotional problems as common among those with EDS, which can lead to avoidance behavior, exacerbation of dysfunction and disability, marginalization, resentment, distrust and hostility between patient, family, and health team. Being ignored, disrespected, and receiving a psychological and/or psychiatric explanation may have consequences for ignoring or not confronting the presence of comorbid psychological problems, resulting in suboptimal treatment. In the same year, Bulbena et al. (48) concluded that there is a growing amount of evidence pointing to the high prevalence of psychiatric conditions among individuals with hEDS.

Other authors have also demonstrated a high prevalence of anxiety disorders, with panic disorder and simple phobia (44, 66). It is evident that anxiety disorders, depression, and neurological development disorders such as attention deficit hyperactivity are, among other clinical manifestations, associated with JH (43, 44).

In this study, among the psychosocial characteristics, fatigue accounted for more than half of the total sample. Clumsy, stubborn, or restless individuals and with attention deficit accounted for just under half of the total sample. Anxiety, stigmatized as “lazy,” insomnia, sleepiness, social isolation, apathy, depression, and impaired spatial and/or temporal orientation represented a minimum of 15.76% and a maximum of 28.01%. In this context, fatigue in hEDS is considered multifactorial (17, 67, 68). It is present in most affected individuals, manifesting with a persistent feeling of tiredness, lack of energy, and feeling of exhaustion, with impaired concentration (51). Increased rates of celiac disease were identified in hEDS, suggesting intestinal malabsorption as a possible factor for fatigue (51, 69). Results of this study on fatigue corroborate the literature, and Bravo (26) found symptoms of chronic fatigue, dizziness, and fainting in 40% of men and 64% of women under 30 years of age. The manifestation of fatigue and musculoskeletal pain, in general, receives little medical attention (52).

Recent studies show manifestation of malaise, lack of concentration and memory, and low self-esteem and self-worth, with a change in the role in society, anxiety, and depression (46), and providing evidence for a longitudinal association between JH and depression at 18 years of age (70). Depressive feelings are common in hEDS and can be understood as secondary to disease-related difficulties such as pain, disability, frustration with the medical system, living a restricted life, etc. (47).

Patients with hEDS often suffer from anxiety disorder, and the link between these two variables has been repeatedly found in the literature (48). It is also associated with a higher frequency and intensity of fears, a higher anxiety severity, greater somatic complaints, and a higher frequency of the so-called endogenous anxiety disorders (49). In 2021, De Vries et al. showed that adolescents and young adults with combined GJH and anxiety were significantly impaired, showing decreased physical and psychosocial functioning with a decreased workload, increased fatigue, and disabling pain. This study confirms the association between GJH and anxiety (50).

In general, people who suffer from EDS report how they must deal with the social image that people around them have, because although they look normal, they have many restrictions that limit their daily routines. Thus, they are labeled lazy, apathetic, and tired people who cannot accomplish tasks, when in fact they are too exhausted to do so (53). In a current qualitative study, participants described internalized negative feelings about their own bodies. They described themselves as “weird” or “useless,” feeling shame and guilt. They found difficulty in keeping up and maintaining contact with friends and family, which generated feelings of frustration and anger among participants, as their joints did not always support their desires. In addition, due to the invisible nature of this disease, they faced judgments from friends, family, and strangers (54).

The transition from the lying to orthostatic position can lead to a state of physical discomfort, with dizziness and fainting, among others, due to lack of oxygen to the brain. Thus, some patients present an excessive resting pattern. Therefore, patients with EDS are often labeled as hyperactive or lazy or still as unsociable and depressed (71).

Shame, guilt, and stigma can have negative psychosocial consequences, decreases self-esteem, and may lead to depression (54). There is anecdotal evidence that symptoms can be triggered by an event, which then has long-term consequences, eventually leading to feelings of isolation (55).

Patients with hEDS report sleeping problems, including insomnia and non-restorative sleep (14, 59). Several factors can interfere with sleep in this population, including pain, dysautonomia, and use of medications (72). Baeza-Velasco et al. (39) cited that reduced sleep is related to the complaint of pain, with psychosocial consequences and chronicity for the quality of life. This study showed that 26 (43.9%) individuals of the total sample reported some sleep disorder. Of these, 16 (28.1%), represented by the majority, reported sleepiness, seven others (12.3%) insomnia, and two (3.5%) daytime sleepiness and nocturnal insomnia. Fibromyalgia is a common concomitant (73, 74) and is strongly associated with sleep disturbances, including abnormal sleep architecture (75). Chronic fatigue and orthostatic intolerance are common in hEDS (76). Hakim and Grahame (14) demonstrated for the first time the significantly increased risk of these symptoms in hEDS. Wandele et al. (77) concluded that autonomic symptoms contribute to the burden of symptoms such as fatigue, dizziness, fainting, syncope, memory, and concentration problems (56–58).

Results in our study showed that pain in the lower limbs was referred by the majority and “growing pain” by more than one third of the total sample, as well as the more the total Beighton score increases, there is a tendency not to have “growing pain.” Matsudo et al. (60) corroborate our results. These authors also identified the lower limbs as the most predominant location for “growing pain.” Early recognition and appropriate treatment of problems such as sleep function, mobility, chronic pain, and psychological conditions are important for successful holistic treatment of patients with EDS (61).

Psychosocial aspects are multifactorial and suggestive of manifestations since childhood, influenced by the composition of less robust tissues, with consequences on body mechanics and motor performance in daily, instrumental, recreational, sports, and occupational activities. Added to these factors, the manifestation of pain since childhood, which together may justify the psychosocial implications, with damage to social inclusion.

## Conclusion

In the total sample, the JH characteristic prevails in the upper limbs of female children, adolescents and adults, with a total Beighton score ≥6. Most sit in the “concave” position and less than half also sit in the “W” position and with atypical hand postures. The higher Beighton scores, which include the upper limbs, show a tendency to not crawl or crawl differently, delayed ambulation, and impaired school performance. The predominance of JH in the upper limbs is suggestive of a justification for not crawling or crawling differently. Characteristics of atypical motor performance in hands and sitting posture, in addition to fatigue, pain since childhood, anxiety, apathy, depression, sleep disorders, stigmatization, attention deficit, spatial and/or temporal orientation impairment, and social isolation are characteristics. suggestive of psychosocial implications at different ages. Future studies with motor and psychosocial aspects of people with JH will help to identify the phenotype of this population and consequent guidance for clinical management based on the motor and psychosocial aspects of people with JH.

## Data Availability Statement

The raw data supporting the conclusions of this article will be made available by the authors, without undue reservation.

## Ethics Statement

The studies involving human participants were reviewed and approved by Comitê de ética em pesquisa—CEP—Faculdade de Medicina de São José do Rio Preto. Written informed consent from the participants' legal guardian/next of kin was not required to participate in this study in accordance with the national legislation and the institutional requirements.

## Author Contributions

All authors listed have made a substantial, direct, and intellectual contribution to the work and approved it for publication.

## Conflict of Interest

The authors declare that the research was conducted in the absence of any commercial or financial relationships that could be construed as a potential conflict of interest.

## Publisher's Note

All claims expressed in this article are solely those of the authors and do not necessarily represent those of their affiliated organizations, or those of the publisher, the editors and the reviewers. Any product that may be evaluated in this article, or claim that may be made by its manufacturer, is not guaranteed or endorsed by the publisher.
